# Association between uterine artery embolization for postpartum hemorrhage and second delivery on maternal and offspring outcomes: a nationwide cohort study

**DOI:** 10.1093/hropen/hoae043

**Published:** 2024-06-26

**Authors:** Woo Jin Yang, Danbee Kang, Ji-Hee Sung, Myung Gyu Song, Hyejeong Park, Taegyun Park, Juhee Cho, Tae-Seok Seo, Soo-Young Oh

**Affiliations:** Department of Radiology, Korea University Guro Hospital, Korea University College of Medicine, Seoul, Republic of Korea; Department of Clinical Research Design and Evaluation, SAIHST, Sungkyunkwan University, Seoul, Republic of Korea; Center for Clinical Epidemiology, Samsung Medical Center, Seoul, Republic of Korea; Department of Obstetrics and Gynecology, Samsung Medical Center, Sungkyunkwan University School of Medicine, Seoul, Republic of Korea; Department of Radiology, Korea University Guro Hospital, Korea University College of Medicine, Seoul, Republic of Korea; Center for Clinical Epidemiology, Samsung Medical Center, Seoul, Republic of Korea; Department of Clinical Research Design and Evaluation, SAIHST, Sungkyunkwan University, Seoul, Republic of Korea; Department of Big Data Research and Development, National Health Insurance Service, Wonju, Republic of Korea; Department of Clinical Research Design and Evaluation, SAIHST, Sungkyunkwan University, Seoul, Republic of Korea; Center for Clinical Epidemiology, Samsung Medical Center, Seoul, Republic of Korea; Department of Radiology, Korea University Guro Hospital, Korea University College of Medicine, Seoul, Republic of Korea; Department of Obstetrics and Gynecology, Samsung Medical Center, Sungkyunkwan University School of Medicine, Seoul, Republic of Korea

**Keywords:** uterine artery embolization, postpartum hemorrhage, preterm birth, major congenital malformations, adverse neonatal events, attention-deficit/hyperactivity disorder

## Abstract

**STUDY QUESTION:**

What are the maternal and neonatal outcomes of second delivery in women who underwent uterine artery embolization (UAE) during their first delivery?

**SUMMARY ANSWER:**

Women who underwent UAE during their first delivery exhibited higher risks of placental problems, preterm births, and postpartum hemorrhage (PPH) in second delivery and the second offspring also showed increased risk of major congenital malformations, admission to the neonatal intensive care units (NICU), necrotizing enterocolitis, intraventricular hemorrhage, and bronchopulmonary dysplasia.

**WHAT IS KNOWN ALREADY:**

UAE is a minimally invasive procedure used as an alternative to hysterectomy for managing severe PPH. However, recent studies have raised concerns about potential obstetric complications, including recurrent PPH, placenta accreta spectrum (PAS), and fetal growth restriction in subsequent delivery following UAE.

**STUDY DESIGN, SIZE, DURATION:**

This was a nationwide retrospective cohort study using the Korean National Health Insurance Service (K-NHIS) database, covering 50 million individuals from 2004 to 2020. The cohort included 3 616 923 women with live births between 1 January 2005 and 31 December 2019 with follow-up data extending to 31 December 2020.

**PARTICIPANTS/MATERIALS, SETTING, METHODS:**

The study included women who had their first live birth between 2005 and 2019, excluding those who underwent hysterectomy (without UAE = 3 612 389, UAE = 4534). Among them, we selected women who had single gestation secondary delivery (without UAE = 1 694 600, UAE = 1146). Propensity score matching was used to control for confounding factors, resulting in 11 184 women without UAE and 1119 women with UAE for subsequent analysis.

**MAIN RESULTS AND THE ROLE OF CHANCE:**

Women in the UAE group had significantly higher risks of PAS (odds ratio (OR) = 38.91, 95% CI = 18.61–81.34), placenta previa (OR = 6.98, 95% CI = 5.57–8.75), and preterm birth (OR = 2.23, 95% CI = 1.71–2.90) during their second delivery. The risk of recurrent PPH was also significantly higher (OR = 8.94, 95% CI = 7.19–11.12). Their second offspring were more likely to have major congenital malformations (OR = 1.62, 95% CI = 1.25–2.11) and adverse neonatal outcomes, including NICU admissions (OR = 1.83, 95% CI = 1.48–2.25). Long-term outcomes showed a higher risk of attention-deficit/hyperactivity disorder (hazard ratio = 1.64, 95% CI = 1.03–2.63) but were otherwise comparable to those in the without UAE group.

**LIMITATIONS, REASONS FOR CAUTION:**

Retrospective nature of the study may have introduced exposure and outcome misclassifications, despite the reliability of the K-NHIS database. Unmeasured confounders and selection bias due to only including live births could also have influenced the results.

**WIDER IMPLICATIONS OF THE FINDINGS:**

Women with a history of UAE require meticulous prenatal care and close monitoring during subsequent deliveries due to increased risks of complications. Counseling and referral to high-risk medical centers may improve outcomes. Further research is needed to understand the mechanisms of complications in both mothers and offspring at sequential delivery, as well as to refine UAE procedures.

**STUDY FUNDING/COMPETING INTEREST(S):**

This study supported by Patient-Centered Clinical Research Coordinating Center (PACEN) funded by the Ministry of Health & Welfare, Republic of Korea (HC21C0123). This study was funded by S.-Y.O. The authors of this manuscript declare no relationships with any companies whose products or services may be related to the subject matter of the article.

**TRIAL REGISTRATION NUMBER:**

N/A.

WHAT DOES THIS MEAN FOR PATIENTS? The purpose of our study was to understand how a procedure called uterine artery embolization (UAE), used to control severe bleeding after childbirth, affects women in their next delivery. We aimed to determine if UAE could lead to more problems in future deliveries and how it might impact the health of their next baby.

Women who had UAE during their first delivery were more likely to experience complications in their next delivery. They had higher risks of placental problems, such as placenta accreta spectrum and placenta previa, and were more likely to give birth prematurely and experience severe bleeding again. Babies born to women who had UAE were more likely to have major birth defects, particularly heart problems. They also had a higher chance of needing special care in the neonatal intensive care unit. Most long-term health problems in children, such as developmental delays or cerebral palsy, were not more common in children born to mothers who had UAE. However, there was a slightly higher risk of attention-deficit/hyperactivity disorder (ADHD) in these children.

Women who have had UAE after their first delivery should have careful and regular check-ups during their next pregnancy. The mother and physician should be aware of the increased risks for both the mother and the baby, including potential complications with the placenta and the possibility of preterm birth. Parents should discuss with the doctor the need for special monitoring and possibly delivering at a high-risk medical center to ensure the best care for the mother and baby. Awareness of these risks can help the mother and healthcare team plan for a safer pregnancy and delivery.

## Introduction

Postpartum hemorrhage (PPH) is defined as cumulative blood loss of ≥1000 ml, or blood loss accompanied by signs of hypovolemia within 24 h after delivery (Bateman *et al.*, 2010; Committee on Practice Bulletins-Obstetrics, 2017). PPH occurs in ∼4–6% of deliveries (Kearney *et al.*, 2018), and severe PPH contributes to ∼140 000 maternal deaths annually worldwide (Matsuzaki *et al.*, 2017). Traditional approaches to managing PPH involve administering a uterotonic drug, uterine compression, and intrauterine balloon tamponade (Committee on Practice Bulletins-Obstetrics, 2017; Chen *et al.*, 2018). When conservative management of PPH fails, uterine artery embolization (UAE) or other surgical management is commonly used to lower the risk of maternal mortality (Committee on Practice Bulletins-Obstetrics, 2017; Chen *et al.*, 2018).

For managing severe PPH, UAE is considered a useful alternative to hysterectomy (Committee on Practice Bulletins-Obstetrics, 2017; Chen *et al.*, 2018), offering an effective and minimally invasive procedure with fewer side effects. Notably, UAE has a consistent success rate of over 90% in achieving hemostasis (Matsuzaki *et al.*, 2021). The fertility rate following UAE for patients attempting another pregnancy ranges from 70% to 80% (Soro *et al.*, 2017). However, a recently growing concern is that UAE may negatively impact subsequent pregnancies in certain cases. Notably, recent systematic reviews and large-scale comparative datasets suggest a potential association between UAE and increased rates of various obstetric complications, such as recurrent PPH and placenta accreta spectrum (PAS) in subsequent deliveries. Additionally, offspring born to women with a history of UAE have shown an elevated increased risk of fetal growth restriction (FGR) (odds ratio (OR) = 7.22, 95% CI = 0.28–188.69) and preterm birth (OR = 3.00, 95% CI = 0.74–12.14), although this evidence is limited by small sample sizes (Matsuzaki *et al.*, 2021).

Most studies examining gynecological and obstetrical adverse outcomes after UAE for PPH have been constrained by limited statistical power due to short-term evaluations or lack of comparison with women who did not undergo UAE. However, the risks of short- and long-term adverse events in subsequent offspring have not been thoroughly investigated. Therefore, we conducted a nationwide cohort study to explore the relationship between UAE during the first delivery and the risks associated with the second delivery and outcomes of the second offspring compared with women who did not undergo UAE during their first delivery.

## Materials and methods

### Data source and study cohort

We conducted a nationwide retrospective cohort study using the Korean National Health Insurance Service (K-NHIS) database, which covered 50 million individuals (∼99% of the South Korean population), from 2004 to 2020 (Kim *et al.*, 2021). The K-NHIS database represents the entire population of South Korea and contains national records of all covered inpatient and outpatient visits, procedures, and prescriptions.

Our cohort included all live births from 1 January 2004 to 31 December 2020 identified with procedure codes specific to delivery. We used 2004 as the washout period, and 2020 as the follow-up period. Thus, we selected the first live delivery from 1 January 2005 to 31 December 2019 (N = 3 618 967, Supplementary Fig. S1). Since our research aimed to evaluate the second delivery outcomes of embolization at the first delivery, we excluded 2044 women who underwent hysterectomy at the first delivery due to their inability to conceive again. Among 3 616 923 women who had experienced their first live birth, 1 694 600 in the non-UAE group, and 1146 in the UAE group had a secondary delivery. We performed 1:10 propensity score matching (PSM) between the without and with UAE groups. Subsequently, the neonatal and long-term outcomes of live births resulting from secondary delivery were compared (Supplementary Fig. S1).

The requirement for informed consent was waived because this study was conducted using anonymized claims data. This study was approved by the Institutional Review Board (2021-08-107).

### Measurement

The K-NHIS data comprise individual-level demographics and all records of diagnosis and healthcare utilization (e.g. drug prescriptions and medical procedures) provided during inpatient, outpatient, and emergency department visits. K-NHIS claims for inpatient and outpatient visits, procedures, and prescriptions were coded using the International Classification of Diseases 10th Revision (ICD-10) (Chun *et al.*, 2009).

The exposed group was women who underwent UAE during the first delivery. PPH usually happens within 1 day of birth; however, it can occur up to 12 weeks after delivery. Therefore, we defined UAE as the presence of the procedure code (M6644) within 2 weeks after birth (UAE group). The unexposed consisted of women who did not undergo UAE at their first delivery, regardless of whether they experienced PPH (without UAE group).

The study endpoints were the maternal outcomes during the second delivery, and major congenital malformations, adverse outcomes, and long-term neurodevelopmental outcomes in the second live offspring. The study participants were followed up until 31 December 2020. A second delivery was defined using procedure codes specific to delivery after the first delivery. Among these, stillbirth (ICD-10: O36) was defined using the ICD-10 code. Maternal events during second delivery were recurrent PPH, Bakri tamponade balloon, UAE, hysterectomy, maternal intensive care unit (ICU) admission. These events were defined as the presence of a disease code or procedure code (Supplementary Table S1) at the second delivery.

Major congenital malformations in second offspring were identified using diagnostic records based on the ICD-10 codes defined by the European Surveillance of Congenital Anomalies classification system (EUROCAT, 2013). The adverse outcome of second offspring was composite morbidity, which included any of the following: transient tachypnea, respiratory distress syndrome, necrotizing enterocolitis, intraventricular hemorrhage, bronchopulmonary dysplasia, and sepsis (Supplementary Table S1). Long-term outcomes of second offspring were defined based on prespecified neurological and neurodevelopmental diagnoses. These included autism spectrum disorder, attention-deficit/hyperactivity disorder (ADHD), cerebral palsy, developmental delay, including motor or cognitive delay, epileptic and febrile seizures and tics, and stereotypic behavior (Supplementary Table S1).

We considered a broad range of covariates as potential confounders or proxies of potential confounders at first deliveries. Maternal age and maternal comorbidities within the year prior to birth were summarized using Charlson’s index (Charlson *et al.*, 1987; Kim, 2010). A history of abortion, including spontaneous abortion, medicolegal abortion, stillbirth, placenta previa, and placental abruption, was also included in the code. A history of uterine surgery and uterine disease was also included using the ICD-10 code or procedure code. We also included comorbidities during pregnancy, including hypertension (ICD-10 codes O14, O11, O15, O13, O16, I10, and O10), gestational diabetes (ICD-10 code O244), and overt diabetes (ICD-10 codes O240, O241, O242, O243, E10, E11, E12, E13, and E14). We defined multiple gestations using the code (ICD-10 code: O30). Owing to the nature of the dataset, no missing values and no losses to follow-up occurred in this study.

### Statistical analysis

In order to minimize the potential confounding factors related to maternal risk, a 1:10 PSM was performed depending on the outcome. For the comparison of second-delivery maternal outcomes, a PSM was performed within only women who had a second delivery with a single gestation. For the comparison of second offspring outcomes, a PSM was also performed from only women who had a single gestation live birth in their secondary delivery. The logistic regression model included maternal age, income level, residence areas, Charlson’s index, history of abortion, stillbirth, uterus surgery, disease of the uterus, hypertension during pregnancy, diabetes during pregnancy, placental problems, and cesarean delivery to estimate the probability of undergoing UAE at first delivery. We implemented nearest-neighbor matching with a caliper of 0.2 on the propensity score (PS) (Austin, 2011). Differences in baseline covariables between the two groups were assessed before and after PSM using an absolute standardized difference with a value of >0.2 indicating a significant difference (Austin, 2011).

For the maternal outcomes at the second delivery, we presented the number of events with the corresponding percentage among the women who had a secondary delivery. We then calculated the OR with a 95% CI using logistic regression to compare the women who had UAE at the first delivery with those who did not. For the neonatal intensive care unit (NICU) admissions, necrotizing enterocolitis, intraventricular hemorrhage, and bronchopulmonary dysplasia of the secondary offspring within 1 year, we also presented the number of events with the corresponding percentage among the second offspring, respectively. Additionally, we calculated the OR with a 95% CI of the offspring in the UAE group to compared to the offspring in the without UAE group. For long-term outcomes, we followed the second offspring from their date of birth until the earliest clinic visit using the disease code, or the last date of our data (December 2020). To determine the incidence rate per 1000 person-years, we divided the number of events by the sum of all follow-up data for study participants. Cox proportional hazards regression models were used to estimate the hazard ratio (HR) with a 95% CI compared to the offspring in the without UAE group. We examined the proportional hazard assumption using plots of the log(–log) survival function and Schoenfeld residuals.

We conducted a sensitivity analysis using data from women who experienced PPH during their first delivery (N = 11 371), comparing the risk of offspring outcomes in subsequent delivery between the without UAE and UAE groups. Additionally, we conducted a quantitative bias analysis based on a probabilistic method to address the impact of unmeasured confounders (Huybrechts *et al.*, 2014). Based on the literature, the live birth probability among unexposed pregnancies without malformation (*P*_00_) was defined as 80% (Lee *et al*., 2019). Furthermore, we assumed the probability of live births among unexposed pregnancies with malformations (*P*_10_) as 60%, based on a previous study (Svensson *et al.*, 2014). Additionally, we considered a 28% lower frequency of live births in the UAE group compared to the general population (Ghanaati *et al.*, 2020). Thus, the probabilities of live births in UAE groups without and with malformations were 52% (*P*_01_) and 32% (*P*_11_), respectively. To account for the difference in the probability of live births between women with and without UAE at first delivery, the corrected relative risks were calculated using the following formula: Corrected relative risk (RR) = Observed RR × (*P*_10_ × *P*_01_/*P*_11_ × *P*_00_).

All analyses were two-sided, and *P*-values <0.05 were considered statistically significant. Statistical analyses were performed using SAS version 9.2 (SAS Institute Inc., Cary, NC, USA) and R software version 3.3.2 (Free Software Foundation Inc., Boston, MA, USA).

## Results

In this study, 3 616 923 women and their liveborn infants were linked with their mothers. Among the first live births without hysterectomy, 4534 (0.12%) women underwent UAE. Compared to the without UAE group, women in the UAE group were older, more likely to have multiple gestational pregnancies, and had a higher prevalence of comorbidities, as well as a higher history of abortion and stillbirth. Additionally, the UAE group was more likely to undergo cesarean delivery than the without UAE group. In the without UAE group, 0.7% had experienced PPH during their first delivery (Supplementary Table S2).

In this study, 1 694 600 patients in the without UAE group during their first delivery had second deliveries, with a rate of 53.1 per 1000-person year. Meanwhile, 1146 women in the UAE group had second deliveries with a rate of 39.9 per 1000-person year. After excluding multiple gestation and 1:10 matching, there were 11 184 and 1119 in the without UAE group and the UAE group, respectively. All the standard mean differences were less than 0.2 as our cutoff in all the characteristics (Table 1).

**Table 1.** Comparison of maternal characteristics in the first delivery of the matched cohort according to the uterine artery embolization of the first delivery.

	Without UAE	UAE	SMD
	(N = 11 184)	(N = 1119)	
**Maternal age (years)**	31.6 (3.6)	31.5 (3.5)	0.013
**Income level**			0.015
Q1 (Lowest)	43 (0.4)	6 (0.5)	
Q2.	2473 (22.1)	242 (21.6)	
Q3.	5795 (51.8)	581 (51.9)	
Q4. (Highest)	2608 (23.3)	263 (23.5)	
Unknown	265 (2.4)	27 (2.4)	
**Rural areas**	4908 (43.9)	492 (44)	0.003
**Charlson’s index, mean (SD)**	0.48 (0.8)	0.49 (0.8)	0.012
**History of abortion**	3115 (27.9)	312 (27.9)	0.009
**History of stillbirth**	123 (1.1)	15 (1.3)	0.019
**History of uterus surgery**	42 (0.4)	6 (0.5)	0.009
**History of disease of uterus**	228 (2)	25 (2.2)	0.016
**Hypertension during pregnancy**	904 (8.1)	84 (7.5)	0.016
**Diabetes during pregnancy**			
Gestational diabetes	2564 (22.9)	282 (25.2)	0.002
Overt diabetes	96 (0.9)	14 (1.3)	0.019
**Placental problems**			
Placenta accrete spectrum	15 (0.1)	6 (0.5)	0.032
Placenta previa	843 (7.5)	82 (7.3)	0.001
Placental abruption	4 (0)	1 (0.1)	0.020
**Cesarean delivery**	4001 (35.8)	390 (34.9)	0.001
**Preterm birth**	216 (1.9)	24 (2.1)	0.032

Values are presented as n (%) or mean (SD).

SMD, standard mean difference; UAE, uterine artery embolization.

During the second delivery, the UAE group had a 38.91-fold higher risk (95% CI = 18.61–81.34) of experiencing PAS and a 6.98-fold higher risk (95% CI = 5.57–8.75) of experiencing placenta previa, compared with the without UAE group (Table 2). The risk of preterm birth was also higher in the UAE group (OR = 2.23, 95% CI = 1.71–2.90) than that of the without UAE group (Table 2). The UAE group also had a much higher risk of PPH in the second delivery than the without UAE group (OR = 8.94, 95% CI = 7.19–11.12, Table 2). Furthermore, the UAE group had a higher risk of receiving a Bakri tamponade balloon (OR = 28.82, 95% CI = 18.73–44.35), embolization (OR = 52.50, 95% CI = 27.40–100.62), and hysterectomy (OR = 5.31, 95% CI = 3.23–8.73) at second delivery than the without UAE group. The UAE group also had higher rates of ICU admission and maternal death during second delivery.

**Table 2.** Result of secondary delivery after first delivery (N = 12 303).

	No. of event (%)		UAE vs No UAE
	Without UAE (N = 11 184)	UAE (N = 1119)	OR (95% CI)
**Stillbirth **	61 (0.5)	9 (0.8)	1.48 (0.73–3.00)
**Fetal growth restriction**	64 (0.6)	9 (0.8)	1.41 (0.70–2.84)
**Placental problems**			
Placenta accreta spectrum	9 (0.1)	34 (3.0)	**38.91 (18.61–81.34)**
Placenta previa	212 (1.9)	133 (10.1)	**6.98 (5.57–8.75)**
Placental abruption	22 (0.2)	5 (0.4)	2.28 (0.86–6.03)
**Preterm birth **	330 (3.0)	71 (6.3)	**2.23 (1.71–2.90)**
**Postpartum bleeding**			
Any postpartum bleeding	202 (1.8)	158 (14.1)	**8.94 (7.19–11.12)**
Bakri tamponade balloon	29 (0.3)	78 (7.0)	**28.82 (18.73–44.35)**
Embolization	11 (0.1)	55 (4.9)	**52.50 (27.40–100.62)**
Hysterectomy	46 (0.4)	24 (2.1)	**5.31 (3.23–8.73)**
**Maternal ICU admission**	82 (0.7)	144 (12.9)	**20.00 (15.13–26.43)**
**Maternal death**	23 (0.2)	3 (0.3)	1.30 (0.39–4.35)

UAE group and without UAE group were matched 1:10 using propensity score. The score was developed using maternal age, income level, residence areas, Charlson’s index, history of abortion, stillbirth, uterus surgery, disease of the uterus, hypertension during pregnancy, diabetes during pregnancy, placental problems, and cesarean delivery. Statistically significant values are shown in bold text.

ICU, intensive care unit; OR, odds ratio; UAE, uterine artery embolization.

After excluding stillbirths, we performed 1:10 matching, resulting in 9625 and 963 second offspring in the without UAE group and the UAE group, respectively. All the standard mean differences were less than 0.2 as our cutoff in all the characteristics at first delivery as well as secondary delivery (Supplementary Table S3). Among 10 888 live offspring from second delivery, the risk of major congenital malformations was 4.6% of 9625 and 7.2% of 963 in the without UAE group and UAE groups, respectively. The OR for major congenital malformations of the second offspring in the UAE group compared to those in the without UAE group was 1.62 (95% CI = 1.25–2.11; Table 3). This was attributed to the higher rate of congenital heart disease diagnosis (Table 3). Considering the difference in the probability of live births between the UAE group and the general population, the corrected relative risk was 1.15. The second offspring in the UAE group were also more likely to be admitted to the NICU within a year (OR = 1.83, 95% CI = 1.48–2.25) and experience adverse neonatal events (OR = 1.31, 95% CI = 1.03–1.66) than those in the without UAE group. Among women who experienced PPH at their first delivery, the OR of the UAE group for major congenital malformations and adverse neonatal outcomes was 1.46 (95% CI = 1.10–1.94) and 1.30 (95% CI = 1.10–1.70), respectively, compared to the without UAE group (Supplementary Table S4). For long-term outcomes during the follow-up (median 10.4 years), the second offspring in the UAE group had a higher HR for ADHD (1.64, 95% CI = 1.03–2.63). Other long-term infant outcomes, including autism, cerebral palsy, developmental delay, and epileptic and febrile seizures were similar between the without UAE group and UAE groups (Table 4). In a sensitivity analysis of offspring from women who experienced PPH at their first delivery, the effect size was found to be comparable to the original results (Supplementary Table S5).

**Table 3.** Neonatal outcomes for live births in second deliveries (N = 10 888).

	No. of event (%)		UAE vs No UAE
	Without UAE (N = 9625)	UAE (N = 963)	OR (95% CI)
**Major congenital malformations**			
Any major congenital malformations	438 (4.6)	69 (7.2)	**1.62 (1.25–2.11)**
Heart defects	291 (3.0)	45 (4.7)	**1.57 (1.14–2.17)**
**Outcomes during a year**			
Neonate intensive care units	690 (7.2)	119 (12.4)	**1.83 (1.48–2.25)**
Composite outcome	640 (6.7)	82 (8.5)	**1.31 (1.03–1.66)**
Sepsis	338 (3.5)	36 (3.7)	1.07 (0.75–1.51)
Transient tachypnea	135 (1.4)	18 (1.9)	1.34 (0.82–2.20)
Respiratory distress syndrome	119 (2.7)	29 (3.1)	**1.47 (1.00–2.18)**
Necrotizing enterocolitis	2 (0.1)	2 (0.2)	**10.01 (1.40–71.16)**
Intraventricular hemorrhage	3 (0.0)	1 (0.1)	**6.68 (1.11–40.00)**
Bronchopulmonary dysplasia	20 (0.2)	6 (0.6)	**3.01 (1.22–7.52)**

UAE group and without UAE group were matched 1:10 using propensity score. The score was developed using maternal age, income level, residence areas, Charlson’s index, history of abortion, stillbirth, uterus surgery, disease of the uterus, hypertension during pregnancy, diabetes during pregnancy, placental problems, and cesarean delivery. Statistically significant values are shown in bold text.

OR, odds ratio; UAE, uterine artery embolization.

**Table 4.** Long-term infant outcomes for live births in second deliveries (N = 10 588).

Long-term outcomes	No. of event (per 1000-person year)		UAE vs no UAE
	Without UAE (N = 9625)	UAE (N = 963)	HR (95% CI)
Autism	55 (0.8)	6 (0.9)	1.22 (0.52–2.83)
ADHD	150 (2.2)	20 (3.3)	**1.64 (1.03–2.63)**
Cerebral palsy	37 (0.5)	4 (0.7)	1.11 (0.40–3.10)
Developmental delay	315 (4.6)	28 (4.7)	0.98 (0.66–1.44)
Epileptic and febrile seizures	702 (10.7)	72 (12.8)	1.07 (0.84–1.36)

UAE group and without UAE group were matched 1:10 using propensity score. The score was developed using maternal age, income level, residence areas, Charlson’s index, history of abortion, stillbirth, uterus surgery, disease of the uterus, hypertension during pregnancy, diabetes during pregnancy, placental problems, and cesarean delivery. Statistically significant values are shown in bold text.

ADHD, attention-deficit/hyperactivity disorder; HR, hazard ratio; UAE, uterine artery embolization.

## Discussion

The UAE group showed higher incidences of placental issues, PPH, and preterm births at second delivery. Additionally, we observed increased risks of overall malformations, particularly heart anomalies, and NICU admissions in the second offspring. However, no significant associations were found between long-term neurological outcomes of the second offspring, such as cerebral palsy or developmental delay, except for ADHD.

In this large national cohort study, the UAE group exhibited lower rates of secondary delivery. This result is similar to the findings of previous studies (Hardeman *et al.*, 2010; Cho *et al.*, 2017). Psychological stress, including sexual dysfunction and fear of another pregnancy due to PPH and UAE, may contribute to the reduced pregnancy rate. Studies have shown that women with higher levels of postpartum posttraumatic stress disorder symptoms at 6-week postpartum are more likely to decide against further pregnancies (Gottvall and Waldenström, 2002). Additionally, reduced desire for pregnancy has been reported in women after UAE compared with those without the procedure (55% vs 93.5%, *P* < 0.001) (Eggel *et al.*, 2021). Therefore, the emotional impact of UAE should be acknowledged, and interventions such as psychological counseling should be considered for affected patients.

Among women with a second delivery, the UAE group had elevated risks of placental issues, including PAS, placenta previa, and placental abruption. This observation may be attributed to the potential damage caused by the use of Gelfoam sponge particles (GSP), commonly used in UAE procedures in Korea. Prolonged inflammation and hypoxia resulting from the temporary embolic effect of GSP could contribute to permanent damage to the arteries (Kim *et al.*, 2023). Furthermore, UAE might affect necrotizing myometritis/endomyometritis and/or acute endometritis, which can potentially lead to the formation of uterine scars (Colgan *et al.*, 2003). Uterine scars probably lack normal architecture and adequate blood supply to meet placental needs during placentation and development (Jing *et al.*, 2018). These histopathologic changes may explain the previous studies suggesting that UAE was associated with placental abnormality in subsequent deliveries, resulting in placental dysfunction and insufficiency (Jitsumori *et al.*, 2020; Eggel *et al.*, 2021; Matsuzaki *et al.*, 2021).

Furthermore, the UAE group demonstrated a higher incidence of recurrent PPH in second deliveries compared with the no-UAE group, consistent with findings from previous studies (Buzaglo *et al.*, 2015; Cho *et al.*, 2017). Uterine damage due to UAE might lead to abnormal placentation, a potential cause of PPH (Salomon *et al.*, 2003). Women in the UAE group may also face an increased likelihood of undergoing procedures, such as Bakri insertion, embolization, and hysterectomy during a second delivery. Although the precise cause of abnormally invasive placenta remains unclear, the prevailing hypothesis suggests a secondary defect in the endometrial–myometrial interface, leading to the failure of normal decidualization (Jauniaux and Jurkovic, 2012). This failure allows deep anchoring of placental villi and infiltration of trophoblasts (Jauniaux and Jurkovic, 2012). Reports of uterine necrosis and uterine synechia after pelvic artery embolization for PPH further highlight the potential contribution of UAE-induced damage to the endometrium and myometrium in the development of abnormally invasive placenta (Imafuku *et al.*, 2020). Our data also revealed a higher rate of maternal ICU admission among women with a history of UAE. Therefore, these women should be counseled regarding the possibility of life-threatening complications. They may benefit from referral to high-risk medical centers for delivery, known to improve outcomes in certain obstetric conditions (Oberg *et al.*, 2014).

We also found that the incidence of preterm birth was higher in the UAE group than in the without UAE group. Placental issues may lead to sequential complications, including preterm birth and PPH. According to a previous meta-analysis investigating the impact of prior UAE on preterm birth during subsequent pregnancies, infants born to women with a history of UAE had 7- and 3-fold increased risks of FGR and preterm birth, respectively (Matsuzaki *et al.*, 2021). Moreover, previous studies had limited power and were insufficient to adequately assess the potential risk of FGR and preterm birth. Importantly, our study had a much larger cohort of pregnancies than any other study published to date, thus expanding on previous findings by providing more precise estimates.

Our investigation revealed that second offspring born to the UAE group faced a higher risk of major congenital malformations, particularly heart disease, compared to those born in the second delivery to the without UAE group. Notably, a high incidence of congenital heart defects was observed, such as patent ductus arteriosus and atrial septal defects among premature infants. We presume that the high incidence of such diagnoses among premature infants in women who underwent UAE during their first delivery was attributable to the higher rate of placenta previa or abruption necessitating preterm birth (Stoll *et al.*, 2010). Additionally, other neonatal adverse events were more prevalent in the UAE group. Therefore, monitoring these women more frequently during pregnancy is necessary considering that previous UAE procedures may increase the risk of poor neonatal outcomes in subsequent delivery.

Regarding long-term outcomes, most neurodevelopmental disabilities were unrelated to previous UAE procedures, except for ADHD. However, the number of events was too small to draw conclusions. Thus, conducting further studies in collaboration with radiologists, obstetricians, and pediatricians is important to investigate the long-term outcomes of infants born to women who have undergone UAE.

Our study had several limitations. First, exposure and outcome misclassifications were possible. However, the data we utilized are widely accepted as reliable and are frequently employed in peer-reviewed publications, as the K-NHIS conducts regular audits of claims (Shin *et al.*, 2016; Lee *et al.*, 2017). Thus, we focused on the delivery outcome which can be captured by procedure code. Furthermore, for UAE, the beneficiaries receive detailed information on claimed items and costs, minimizing the likelihood of missed claims (Noh *et al.*, 2022). Notably, the sensitivity and specificity of ICD-10 codes for identifying PPH were reported to be 88.5% (95% CI = 88.2–88.7) and 99.4% (95% CI = 99.4–99.4), respectively (Ladfors *et al.*, 2021). Similarly, placental problems had a sensitivity of 88% (95% CI = 76–96) and a specificity of 97% (95% CI = 91–99) (Jotwani *et al.*, 2024). Second, our results could have been influenced by unmeasured confounders despite adjusting for numerous confounding factors. However, detailed procedure-related factors were not adjusted for, as we compared women with and without UAE. To mitigate this, we included preterm birth and FGR in the first delivery, which are well-known for their recurrent risk, as covariates in the multivariable analysis (Kalengo *et al.*, 2020). Furthermore, we performed a quantitative bias analysis using the probabilistic method, confirming a higher risk in patients with UAE during their first delivery compared with those without. Moreover, many maternal health factors, such as infertility treatment, were highly correlated with maternal age and history of abortion, stillbirth, uterine surgery, or uterine disease, all of which were already adjusted for. Therefore, we believe that our results are robust. Third, our findings may have been affected by confounding indications, since we used the unexposed group as the reference group. However, when we restricted the study cohort to women with PPH, the results remained consistent with the primary findings. Lastly, our study cohort included only live births, potentially introducing selection bias as severe malformations leading to pregnancy termination would not have been captured. However, we performed PSM depending on the outcome.

According to this nationwide cohort study, women who underwent UAE during their first delivery had a higher likelihood of developing several PPH-associated complications and related preterm births. Most adverse neonatal outcomes were linked to preterm births, potentially influenced by factors like placenta previa and abruption. However, the long-term outcomes of these infants were comparable to those of infants born to women without UAE at first delivery. Thus, women with a history of UAE require meticulous prenatal care from the beginning of their pregnancies, necessitating close collaboration between obstetricians and pediatricians. Additionally, these women require counseling about potential complications and referral to a high-risk medical center for delivery to improve outcomes. Further research is needed to understand the mechanisms of complications in both maternal and offspring at sequential delivery, as well as to refine UAE procedures.

## Supplementary Material

hoae043_Supplementary_Tables

hoae043_Supplementary_Figure_S1

## Data Availability

The data used in this study were approved by the Korean National Health Insurance Service (K-NHIS). The data underlying this study are sourced from the K-NHIS database, which covers ∼99% of the South Korean population. Due to data privacy regulations, the data are not publicly available. However, researchers can request access to the K-NHIS database through the National Health Insurance Data Sharing Service, subject to approval. Further details on data access policies and application procedures can be found at K-NHIS Data Sharing Service (https://nhiss.nhis.or.kr).
