# Supplementary material for: Association between uterine artery embolization for postpartum hemorrhage and second delivery on maternal and offspring outcomes: a nationwide cohort study
Source: Hum Reprod Open. 2024 Jun 26;2024(3):hoae043. doi: 10.1093/hropen/hoae043 (PMC11259214; doi:10.1093/hropen/hoae043)
Supplement: hoae043_Supplementary_Tables [file hoae043_supplementary_tables.docx]

**Supplementary Table S1. Definition of study outcome**

|  | **Codes** |
| --- | --- |
| **1) Delivery** |  |
| Delivery | R3131–R3148, R4351–R4362, R4380, R4507-–R4520, R5001-–R5002, RA311-–RA318, RA361-–RA362, and RA380-–RA434 |
| **2) Outcomes** |  |
| **Maternal outcome** |  |
| Fetal growth restriction | O36.5 |
| Any postpartum bleeding | O22 |
| Bakri tamponade balloon | R4028 |
| Recurrent embolization | M6644 |
| Hysterectomy | R4147, R4148, R4507, R4508, R4509, R4510, R5001, R5001 |
| Placenta accreta spectrum | O43 |
| Placenta previa | O44, O69.4 |
| Placental abruption | O45 |
| Maternal ICU admission | AJ100-–AJ590900 |
| **Major congenital malformations** |  |
| Nervous system | Q00-–Q07 |
| Eye | Q100, Q104, Q106-–Q109, Q11-–Q12, Q130-–Q134, Q136-–Q139, and Q14-–Q15 |
| Ear, face, and neck | Q16, Q176-–Q178, Q183, and Q188 |
| Heart defects | Defects of cardiac chambers and connections (Q20); cardiac septal defects (Q21); pulmonary and tricuspid valve defects (Q22); aortic and mitral valve defects (Q23); other heart defects (Q24); defects of the great arteries (Q25); and defects of the great veins (Q260, Q262-–Q269) |
| Respiratory system | Q300, Q321-–Q329, Q330, Q332-–Q335, Q337-–Q339, and Q34 |
| Oral clefts | Q35-–Q37 |
| Digestive system | Tongue, mouth, and pharynx (Q380, Q383-–Q389); oesophagus (Q39); upper alimentary tract (Q402-–Q409); small intestine (Q41); large intestine (Q42); other malformations of the intestine (Q431-–Q439); gallbladder, bile ducts and liver (Q44); other malformations of the digestive system (Q45); and diaphragmatic hernia (Q790) |
| Abdominal wall defects | Q792-–Q793 and Q795 |
| Urinary system | Q60, Q611-–Q619, Q620-–Q626, Q628-–Q629, Q630-–Q632, Q634-–Q639, Q64, and Q794 |
| Genital organs | Q50-–Q51, Q520-–Q522, Q524, Q526, Q528-–Q529, and Q54-–Q56 |
| Limb | Q660-–Q661, Q679, Q681-–Q682, Q686-–Q689, and Q70-–Q74 |
| Other malformations | Q750, Q77, Q782-–Q788, Q80-–Q81, Q820-–Q824, Q826-–Q829, Q860, Q890, and Q893-–Q894 |
| **Short-term outcome** |  |
| Neonate intensive care units | AJ111, AJ211, AJ311, AJ121, AJ221, AJ321, AJ101, AJ201, AJ301, AJ131, AJ231, AJ331, AJ141, AJ241, AJ341, AJ144, AJ244, AJ301, AJ251, AJ351, AJ161, AJ161, AJ261 |
| Sepsis | R65, A40, A41, O753 |
| Transient tachypnea | P221 |
| Respiratory distress syndrome | P220, P228, P229 |
| Necrotizing enterocolitis | P77 |
| Intraventricular hemorrhage | P520, P521, P522 |
| Bronchopulmonary dysplasia | P271 |
| **Long-term outcome** |  |
| Autism | F84 |
| Cerebral palsy | G80 |
| Developmental delay | F82, R26, R27, F70, F71, F72, F73, F78, F79, F83, F800, F801, F802, F808, F809, F810, F811, F812, F813, F818, F819, R480, R488 |
| Motor developmental delay | F82, R26, R27 |
| Cognitive developmental delay | F70, F71, F72, F73, F78, F79, F83, F800, F801, F802, F808, F809, F810, F811, F812, F813, F818, F819, R480, R488 |
| Attention deficit hyperactivity disorder | F88, F89, F840, F841, F844, F845, F848, F849, F900, F901, F902, F908, F909 |
| Tics and stereotypic behavior | R25, F950, F951, F952, F958, F959, F984, F985 |
| Epileptic and febrile seizures | G40, G41, R56, G253 |

**Supplementary Table S2. Comparison of maternal characteristics in delivery by uterine artery (UAE) embolization before matching (N =** **3,616,923)**

|  | **Without UAE** | **UAE** |  |
| --- | --- | --- | --- |
|  | **(N=3,612,389)** | **(N=4,534)** |  |
| **Maternal age (years)** | 31.9 (4.3) | 34.1 (4.4) |  |
| **Income level** |  |  |  |
| Q1 (Lowest) | 20,355 (0.6) | 30 (0.7) |  |
| Q2. | 793,967 (22.0) | 881 (19.4) |  |
| Q3. | 1,812,700 (50.2) | 2208 (48.7) |  |
| Q4. (Highest) | 896,196 (24.8) | 1298 (28.6) |  |
| Unknown | 89,171 (2.5) | 117 (2.6) |  |
| **Rural areas** | 1,662,837 (46.0) | 1988 (43.8) |  |
| **Charlson’s index, mean (SD)** | 0.5 (0.8) | 0.6 (0.9) |  |
| **History of** **abortion** | 709,213 (19.6) | 1399 (30.9) |  |
| **History of stillbirth** | 18,245 (0.5) | 55 (1.2) |  |
| **History of uterus surgery** | 9,685 (0.3) | 27 (0.6) |  |
| **History of disease of uterus** | 45,283 (1.3) | 167 (3.7) |  |
| **Hypertension during pregnancy** | 119,782 (3.3) | 425 (9.4) |  |
| **Diabetes during pregnancy** |  |  |  |
| Gestational diabetes | 383,609 (10.6) | 661 (14.6) |  |
| Overt diabetes | 37,266 (1.0) | 86 (1.9) |  |
| **Placental problems** |  |  | |
| Placenta accrete spectrum | 945 (0.03) | 22 (0.5) | |
| Placenta previa | 25,035 (0.7) | 482 (10.6) | |
| Placental abruption | 3,763 (0.1) | 21 (0.5) | |
| **Cesarean delivery** | 1,497,417 (41.5) | 2291 (50.5) |  |
| **Preterm birth** | 123,192 (3.4) | 607 (13.4) |  |
| **Multiple gestation** | 68,275 (1.9) | 442 (9.7) |  |
| **Postpartum hemorrhage** | 25,187 (0.7) | 4534 (100) |  |

Values are presented as n (%) or mean (SD).

UAE, uterine artery embolization

| **Supplementary Table S3.** **Comparison of maternal characteristics of matched cohort at second delivery by uterine artery embolization at first delivery among women with a second live birth (N = 10,588)** | | | |
| --- | --- | --- | --- |
|  | **Without UAE** | **UAE** | **SMD** |
|  | **(N= 9,625)** | **(N= 963)** |  |
| **Maternal age (years)** | 34.6 (3.6) | 34.6 (3.5) | <0.001 |
| **Income level** |  |  |  |
| Q1 (Lowest) | 21 (0.2) | 5 (0.5) | 0.050 |
| Q2. | 1,399 (14.5) | 143 (14.8) | 0.009 |
| Q3. | 4,027 (41.8) | 396 (41.1) | -0.015 |
| Q4. (Highest) | 3,753 (39) | 377 (39.1) | 0.003 |
| Unknown | 425 (4.4) | 42 (4.4) | -0.003 |
| **Rural areas** | 3,973 (41.3) | 395 (41) |  |
| **Charlson’s index, mean (SD)** | 0.7 (1) | 0.71 (0.9) | 0.011 |
| **History of abortion** | 3,909 (40.6) | 391 (40.6) | <0.001 |
| **History of stillbirth** | 126 (1.3) | 14 (1.5) | 0.012 |
| **History of uterus surgery** | 16 (0.2) | 4 (0.4) | 0.046 |
| **History of disease of uterus** | 21 (0.2) | 3 (0.3) | 0.018 |
| **Hypertension during pregnancy** | 650 (6.8) | 60 (6.2) | -0.021 |
| **Diabetes during pregnancy** |  |  |  |
| Gestational diabetes | 3,588 (37.3) | 362 (37.6) | 0.006 |
| Overt diabetes | 299 (3.1) | 27 (2.8) | -0.018 |
| **Cesarean delivery** | 5,679 (59.0) | 563 (58.5) | -0.011 |

Values are presented as n (%) or mean (SD).

SMD, standard mean difference; UAE, uterine artery embolization

**Supplementary Table S4. Second offspring outcomes by embolization at first delivery among offspring from women who experienced postpartum bleeding at first delivery (N=11,371)**

| **Short-term outcomes** | **Without UAE** | **UAE** | **Adjusted** |
| --- | --- | --- | --- |
|  | **No. of event (%)** | | **OR (95 % CI)** |
| **Major congenital malformations** | 347 (3.3) | 58 (5.9) | **1.46 (1.10-–1.94)** |
| **Composite outcome during a year** | 409 (3.9) | 81 (8.2) | **1.30 (1.10-–1.70)** |

OR, odds ratio; UAE, uterine artery embolization

Model adjusted for maternal age, income, Charlson's index, history of abortion, stillbirth, placental previa, placental abruption, hypertensive disorder during pregnancy, gestational diabetes, overt diabetes, twin, preterm birth, and fetal growth retardation.

**Supplementary Table S5. Long-term infant outcomes for live births in second deliveries among offspring from women who experienced postpartum bleeding at first delivery (N=11,371)**

| **Long-term outcomes** | **Without UAE** | **UAE** | **Adjusted** |
| --- | --- | --- | --- |
|  | **No. of event (per 1,000-person year)** | | **HR (95 % CI)** |
| **Autism** | 54 (0.7) | 7 (1.1) | 1.51 (0.69-3.33) |
| **ADHD** | 195 (2.5) | 21 (3.4) | 1.43 (0.91-2.25) |
| **Cerebral palsy** | 17 (0.2) | 4 (0.6) | 2.56 (0.86-7.57) |
| **Developmental delay** | 324 (4.3) | 28 (4.6) | 1.02 (0.70-1.51) |
| **Epileptic and febrile seizures** | 723 (9.9) | 73 (12.6) | 1.12 (0.88-1.43) |

HR, hazard ratio; UAE, uterine artery embolization

Model adjusted for maternal age, income, Charlson's index, history of abortion, stillbirth, placental previa, placental abruption, hypertensive disorder during pregnancy, gestational diabetes, overt diabetes, twin, preterm birth, and fetal growth retardation.
