# Supplementary material for: Association between uterine artery embolization for postpartum hemorrhage and second delivery on maternal and offspring outcomes: a nationwide cohort study
Source: Hum Reprod Open. 2024 Jun 26;2024(3):hoae043. doi: 10.1093/hropen/hoae043 (PMC11259214; doi:10.1093/hropen/hoae043)
Supplement: hoae043_Supplementary_Figure_S1 [file hoae043_supplementary_figure_s1.pdf]

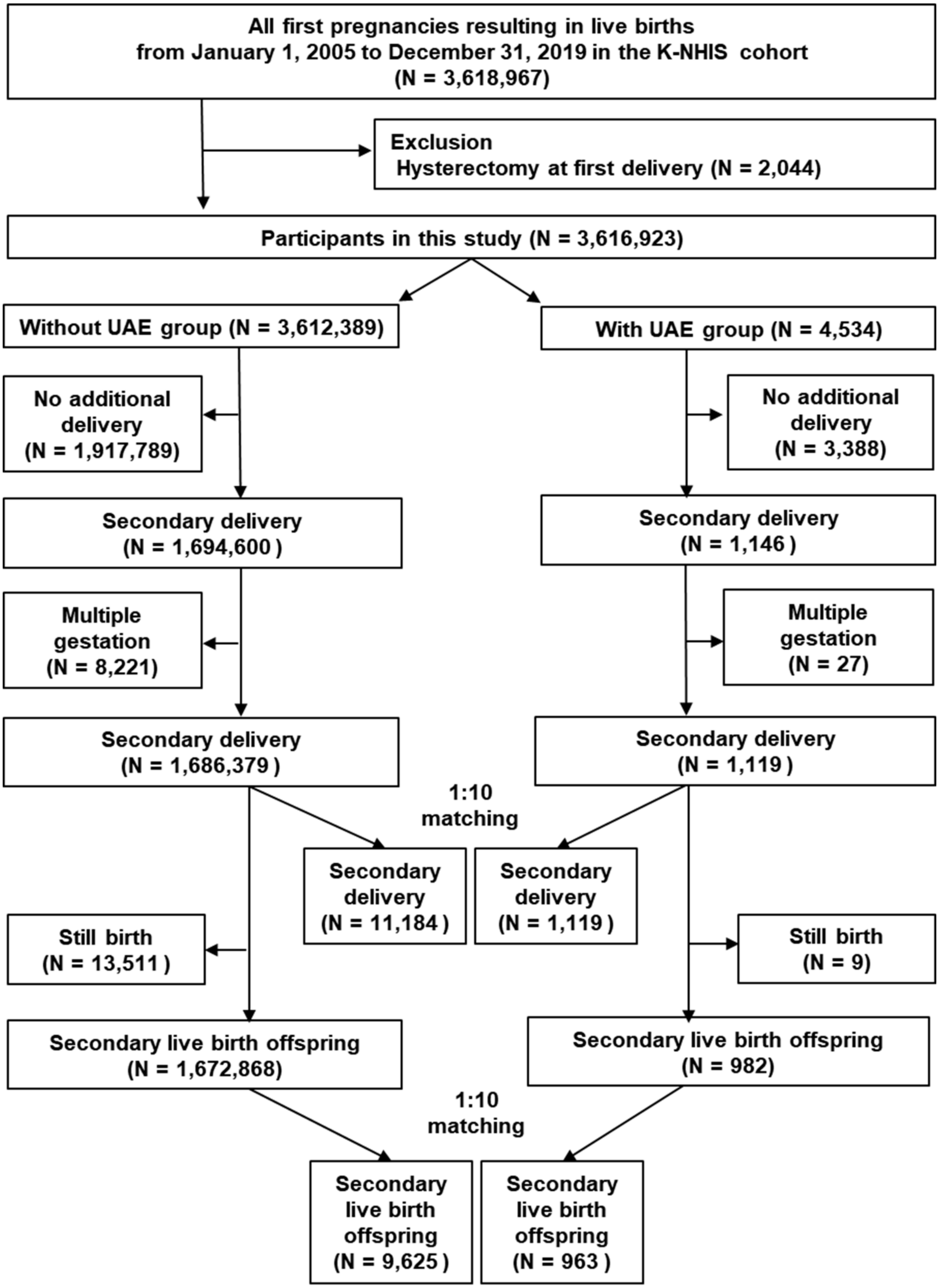

Supplementary Figure S1. Flow diagram showing derivation of study cohort

K-NHIS, Korean National Health Insurance Service ; UAE, uterine artery embolization
